# Soluble L1CAM promotes breast cancer cell adhesion and migration *in vitro*, but not invasion

**DOI:** 10.1186/1475-2867-10-34

**Published:** 2010-09-15

**Authors:** Yupei Li, Deni S Galileo

**Affiliations:** 1 Department of Biological Sciences, University of Delaware, Wolf Hall, Newark, DE 19716 USA

## Abstract

**Background:**

Neural recognition molecule L1CAM, which is a key protein involved in early nervous system development, is known to be abnormally expressed and shed in several types of cancers where it participates in metastasis and progression. The distinction of L1CAM presence in cancerous vs. normal tissues has suggested it to be a new target for cancer treatment. Our current study focused on the potential role of soluble L1CAM in breast cancer cell adhesion to extracellular matrix proteins, migration, and invasion.

**Results:**

We found L1 expression levels were correlated with breast cancer stage of progression in established data sets of clinical samples, and also were high in more metastatic breast cancer cell lines MDA-MB-231 and MDA-MB-435, but low in less migratory MDA-MB-468 cells. Proteolysis of L1 into its soluble form (sL1) was detected in cell culture medium from all three above cell lines, and can be induced by PMA activation. Over-expression of the L1 ectodomain in MDA-MB-468 cells by using a lentiviral vector greatly increased the amount of sL1 released by those cells. Concomitantly, cell adhesion to extracellular matrix and cell transmigration ability were significantly promoted, while cell invasion ability through Matrigel™ remained unaffected. On the other hand, attenuating L1 expression in MDA-MB-231 cells by using a shRNA lentiviral vector resulted in reduced cell-matrix adhesion and transmigration. Similar effects were also shown by monoclonal antibody blocking of the L1 extracellular region. Moreover, sL1 in conditioned cell culture medium induced a directional migration of MDA-MB-468 cells, which could be neutralized by antibody treatment.

**Conclusions:**

Our data provides new evidence for the function of L1CAM and its soluble form in promoting cancer cell adhesion to ECM and cell migration. Thus, L1CAM is validated further to be a potential early diagnostic marker in breast cancer progression and a target for breast cancer therapy.

## Background

Cell adhesion and migration are fundamental processes that occur during organogenesis, neural development, tissue regeneration and immune response, all of which require communication between cells and interaction of cells with their microenvironment. These two processes are also critical for tumor cells traveling to distant sites during metastasis [1,2]. Recent studies on molecules involved in cancer metastasis have found that several neural cell recognition molecules are abnormally expressed and functioning in clinical patient samples and in *in vitro *tumor models [3-5]. Surface proteins, such as N-CAM [6,7], Ng-CAM [8], L1CAM [9,10] and neogenin [11], which predominantly exert effects in nervous system development, have been demonstrated also to facilitate tumor cell progression in certain types of cancer.

L1CAM (CD171) is the initial member of the L1 family of immunoglobulin superfamily proteins and has pivotal roles in mediating the correct formation of neuronal connections during embryo neurogenesis [12-14]. L1 and its homologous cell adhesion molecules are distributed mainly in the central and peripheral nervous systems. With six Ig-like and five fibronectin type III (FN III) domains in the extracellular region and a conserved intracellular cytoplasmic tail, this transmembrane glycoprotein possess sufficient functioning domains to interact with guiding cues or extracellular matrix proteins. Such interactions of neurons with their immediate environment instruct cell and/or axonal movement [15]. L1CAM performs important functions in neuron-neuron adhesion, neuronal migration, neurite extension and fasciculation, axon outgrowth and synaptic plasticity [5,13,15-17]. The importance of L1CAM in the nervous system can be emphasized by the severe syndromes that result from various L1 gene mutations categorized as L1-syndrome [12,13,18-20]. One of the most severe results of this syndrome is hydrocephalus, which in many cases is due to mutations causing production of truncated L1 ectodomain, which is secreted. On the other hand, Kalus et al. [21] found that L1-dependent neurite outgrowth requires highly regulated proteolysis of L1 at the cell surface. Otherwise the cellular microenvironment would be unfavorable for axon outgrowth. These findings shed some light on the potential role of L1 proteolytic cleavage and release of soluble L1 in facilitating neuron migration and axon growth cone protrusion in the nervous system.

Normally, non-neuronal expression of L1 can be found only in Schwann cells in the peripheral nervous system, in some lymphocytes and in part of the renal system [22,23]. But recent studies unveiled L1CAM's abnormal presence in glioma, melanoma, ovarian, colon and pancreatic cancers [4,10,24,25]. According to these studies, tumor cells tend to use the same mechanism involving L1 as neurons do in brain development to possess an increased migratory ability. Proteolysis of L1, however, is not tightly regulated as it is in the nervous system, and is constitutive. The abnormal expression of L1 in cancerous tissues compared to the normal tissues suggests its potential as a marker for tumorigenesis.

Soluble L1 resulting from ADAM10 cleavage has been found in serum and ascites fluid from patients with ovarian carcinoma or endometriosis [26-28]. This proteolyzed form is proposed either to *cis*-interact with integrin αvβ3, αvβ5 [29] on the cell surface, or to become integrated into the immediate extracellular matrix [30]. Studies from other research groups [27,31] and ours [32] have shown that in colon cancer and glioma models, L1CAM expression and proteolysis can be detected at the invasive front of cell cultures, indicating the role of L1 shedding in the pioneering stage of cancer cell migration. Expression and cleavage of L1 in breast cancer cells can be detected both directly from the cell surface and from secretory vesicles [9,30]. Whether this soluble form can facilitate breast cancer cell migration or have any roles in other steps of metastasis is still unclear.

In view of previous studies, our current work aims at elucidating the function of L1CAM, especially its soluble form, in breast cancer cell migratory processes. From established datasets on clinical samples of breast cancer, L1 is found to be among the top ranking of genes whose expression is up-regulated, and expression is high in cells with active migration ability *in vitro*. Over expression of L1 ectodomain alone is adequate to increase cell adhesion to ECM and increase transmigration, and this effect can be blocked with anti-L1CAM antibody. We provide novel evidence to show the soluble form of L1 can be a chemotactic signal to direct cell migration. These results further emphasize the potential of L1CAM to be a target for breast cancer treatment.

## Results

### L1CAM expression level increases with breast cancer cell progression

**Figure 1:**
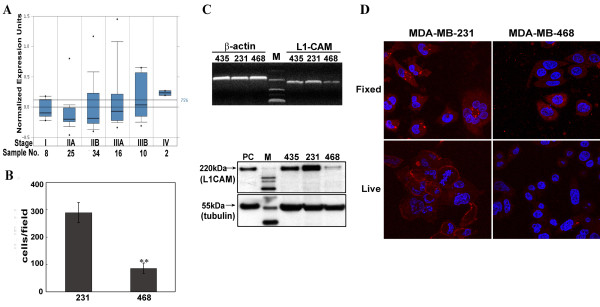
**Characterization of L1CAM expression in human breast cancer**. (A) ONCOMINE gene microarray database was explored for L1CAM gene expression in breast cancer and the results of Boersma et al. [33] were displayed by different stages. COPA analysis yielded a score at 1.95, ranking L1CAM 125^th ^at 75% outlier among 12,427 measured genes on mRNA level (http://www.oncomine.org/). (B) Quantitative analysis of transmigration assay between MDA-MB-231 and MDA-MB-468 cells. Each cell suspension (10^5 ^cells) was plated into the upper chamber of Transwell inserts precoated with LN on the underside, and allowed to migrate for 12 h at 37°C. Cells that migrated to the backside of the insert were stained with crystal violet and counted from five random views. Data are mean ± SEM of three independent experiments. **, p < 0.01. (C) L1CAM expression level examined by RT-PCR and western blot analysis in MDA-MB-435, MDA-MB-231 and MDA-MB-468 cells. Beta-actin and β-tubulin were internal controls for each assay respectively. DNA band markers and protein molecular weight markers are shown as indicated. (D) Immunostaining of L1CAM in MDA-MB-231 and MDA-MB-468 cells observed by confocal microscopy. Fixed and permeablized staining with polyclonal antibody NCAM-L1 (C-20) (upper panel) or live staining with monoclonal antibody UJ127 for L1CAM (lower panel) are shown in red. Nuclei stained with bisbenzimide are shown as blue.

Based on the size and invasiveness of tumors, cancer can be categorized into several advancing stages. To determine whether L1CAM expression level is correlated with breast cancer progression, we used Oncomine (http://www.oncomine.org) to examine microarray results obtained by Boersma et al. [33] on patients with different stages of breast cancer. As shown in Figure 1A, COPA outlier analysis on mRNA levels of 12,427 measured genes from 95 samples identified L1CAM among the top 2% genes that are over-expressed, ranking it 125 at 75th percentile and 164 at the 90th percentile threshold. Also, the percentage of patients with high level L1CAM expression was shown to increase with the advance of cancer progression. In our work here, analysis of L1CAM expression in three breast cancer cell lines, MDA-MB-435, MDA-MB-231 and MDA-MB-468, revealed that MDA-MB-468 cells showed relatively low L1CAM expression both at mRNA and protein levels compared to the other two (Figure 1C). Consistent with results of others [34], MDA-MB-468 also was the less migratory cell line in our transmigration assay (Figure 1B).

Cellular localization of L1CAM by confocal microscopy was apparent on the cell surface, especially at cell-cell contacts in MDA-MB-231 cells after live immunostaining analysis (Figure 1D). Whereas, distributed punctate stained vesicles were observed in the cytoplasm of fixed cells. MDA-MB-468 cells showed low L1CAM expression in both cases.

### Detection and activation of L1 shedding in breast cancer cells

**Figure 2:**
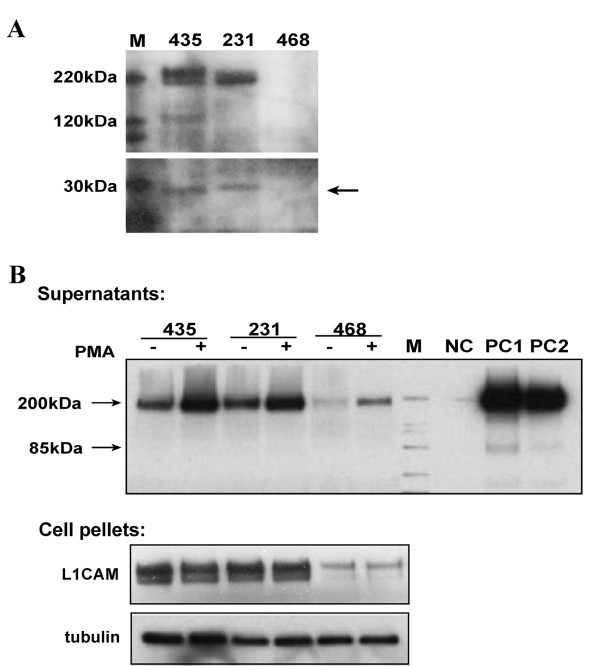
**L1 proteolysis in breast cancer cell lines**. (A) Western blot analysis of L1CAM expression in MDA-MB-231, MDA-MB-435 and MDA-MB-468 cells. 30 μg extracts from each cell line were probed with NCAM-L1 (C-20) for L1. A fragment around 30 kDa after cleavage is shown (arrow) in MDA-MB-435 and MDA-MB-231 cell extracts. (B) Activation of L1 shedding by PMA. Confluent cells after one day in culture were incubated at 37°C for 1 hr with or without 100 ng/mL PMA. Cell culture supernatants were then collected and TCA precipitated, and cell pellets were lysed respectively. Monoclonal antibody UJ127 was used to probe L1 in all samples. NC, plain QT6 cells as negative control. PC1 and PC2, glioma cell lines U-87 MG and T98G as positive controls.

The 200-220 kDa cell surface L1CAM protein can be cleaved by plasmin in the third FNIII domain, releasing 140 kDa and 80 kDa fragments [35]. It also can be proteolyzed by ADAM10 at the membrane proximal end of the extracellular domain into a soluble fragment of approximately 180-200 kDa and a remaining membrane bound fragment of about 30 kDa, which could be further degraded by γ-secretase [21]. We examined L1 cleavage in our cells first by probing the whole cell lysates with antibody NCAM-L1(C-20) against the C-terminus of the encoded protein. The full-length (200-220 kDa), ectodomain fragment (180 kDa) and transmembrane fragment (30 kDa) was detected in MDA-MB-435 and MDA-MB-231 cells (Figure. 2A). MDA-MB-468 cell lysates showed a very low level of L1 protein expression overall. This suggested that our breast cancer cells proteolyzed L1CAM via ADAM10 to release the large ectodomain fragment. We then checked for the presence of soluble L1 in cell culture medium by TCA precipitation. Phorbol ester (PMA) stimulation was reported to increase L1 shedding by ADAM10 [9,30]. As seen in Figure 2B, PMA treatment resulted in a significant increase of soluble L1 (sL1) at 180 kDa in the culture medium compared to samples prior to treatment. Even in MDA-MB-468 cell culture supernatant, a clear band of sL1 could be detected after treatment. However, no apparent changes occurred in the amount of total L1CAM in PMA activated cell pellets, indicating this PMA treatment only activated L1 proteolysis but did not affect overall L1 protein expression levels.

### Over-expressing L1-ectodomain in MDA-MB-468 cells promoted cell adhesion and migration, but not invasion

**Figure 3:**
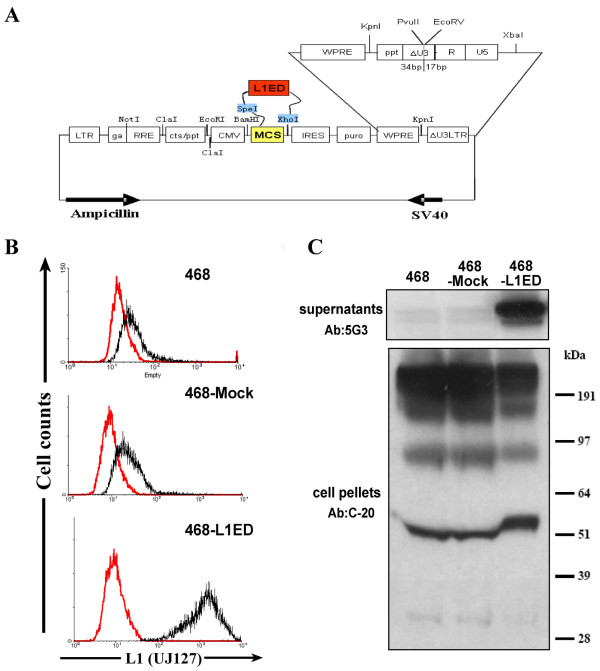
**Over-expressing L1-ectodomain in MDA-MB-468 cells**. (A) Schematic diagram of Lvv 1879 vector containing L1ED. 3350 bp L1 ectodomain fragment was amplified and inserted into Lvv 1879 via SpeI and XhoI restriction enzyme sites. The constructed lentivirus was used to infect MDA-MB-468 cells to establish a new stable cell line. (B) Immunostaining and FACS analysis of L1CAM level in MDA-MB-468-L1ED compared to mock vector infected and plain MDA-MB-468 cells. (C) TCA precipitation and western blotting examining over-expressed L1 ectodomain release in MDA-MB-468-L1ED culture medium by monoclonal antibody 5G3. The amount of cell associated L1 in pellets was probed by polyclonal antibody NCAM-L1 (C-20).

To investigate whether the higher migratory ability of MDA-MB-231 cells compared to MDA-MB-468 (Figure 1B) was due to a higher level of L1 expression and shedding, we established a stable cell line MDA-MB-468 cells over-expressing the L1 ectodomain (MDA-MB-468-L1ED) by lentiviral vector infection (Figure 3A). As shown in Figure 3B and 3C, the infected 468-L1ED cells successfully displayed a high level of L1 protein expression as detected by UJ127 antibody using FACS analysis (Figure 3B), and the over-expressed soluble L1ED was released into the cell culture medium as confirmed by western blot analysis (Figure 3C).

**Figure 4:**
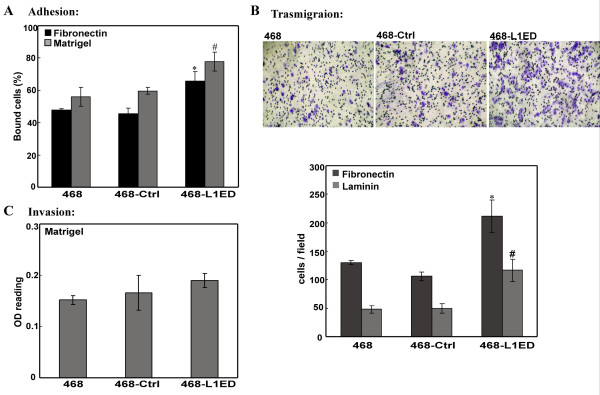
**Increased L1ED expression promotes cell adhesion and transmigration**. (A) Plain MDA-MB-468, mock vector- or L1ED-infected cells were seeded into 96-well plates pre-coated with Matrigel™ (grey bar) at 20 μg/mL or fibronectin (black bar) at 10 μg/ml for the cell adhesion assay as described. (B) Cell transmigration assay using different MDA-MB-468 cell types. Shown are representative undersides of membranes stained with crystal violet after transmigration towards fibronectin. Quantitative analysis of cells migrated to the underside of the inserts is summarized in the lower panel. Data are mean ± SEM of three independent experiments, each with triplicate sets. * and #, P < 0.05, vs. mock vector. (C) Cell invasion analysis on Matrigel™ transwell inserts coated on the top side.

We then tested any change of cell adhesion and transmigration ability in the new stable MDA-MB-468 cell line over-expressing L1ED. In short, L1ED over-expression promoted cell adhesion to fibronectin and Matrigel™. Shown in Figure 4A, after 45 min incubation, compared with plain MDA-MB-468 cells and those infected with empty vector as control, MDA-468-L1ED cells resulted in a significant increase in adhesion of about 20% to fibronectin and 15% to Matrigel™. Similarly in the transmigration assay, almost two fold more cells migrated to the underside of transwell inserts coated with fibronectin or laminin for MDA-MB-468-L1ED cells, while the control vector infected cells showed the same migration rate as uninfected MDA-MB-468 cells (Figure 4B). However, no significant difference was observed for MDA-MB-468-L1ED cells in the invasion assay with transwell inserts coated on top with Matrigel™ (Figure 4C).

### Attenuating L1 expression in MDA-MB-231 cells decreased adhesion and transmigration

**Figure 5:**
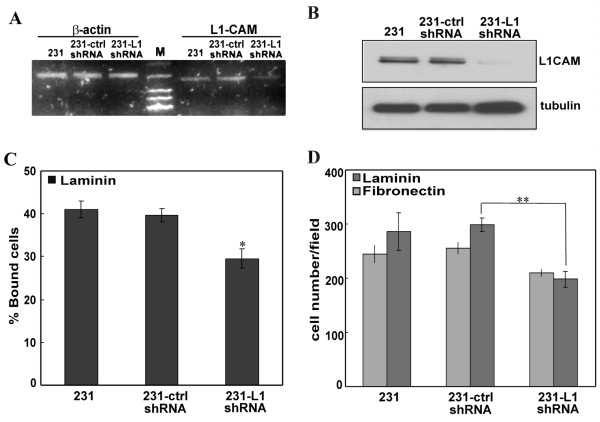
**Attenuating L1CAM in MDA-MB-231 decreases cell adhesion and migration**. Efficiency of L1CAM-shRNA to knock down L1 expression in MDA-MB-231 cells was examined by rtPCR (A) and western blot (B). (C) Analysis of cell adhesion after L1-shRNA infection. Assays were done as previously described. (D) Cell transmigration assay was done as described previously on laminin- or fibronectin-coated transwell inserts. Results shown are mean ± SEM of three independent experiments with triplicates. **, P < 0.01 vs MDA-MB-231 cells infected with control vector.

With the above findings that soluble L1 facilitates cell adhesion and migration, we then tested the effect of attenuating L1 expression by shRNA on breast cancer cell motility. A commercially available shRNA vector targeting L1 was used previously by our group [36]. Here, the attenuating effect of L1-shRNA in MDA-MB-231 cells was shown by RT-PCR and western blot in Figure 5A and 5B. Though slightly detectable L1 remained, over 90% of L1CAM was knocked down after shRNA interference. As seen in Figure 5C, a significant decrease (12% less) of cell adhesion to laminin was obtained for MDA-MB-231 cells infected with L1-shRNA compared to the control infected or uninfected cells. A dramatic (30%) drop also was detected in the number of transmigrated cells for MDA-MB-231-L1shRNA in the transmigration assay onto laminin (Figure 5D). A less dramatic but significant drop in transmigration (16%) occurred onto fibronectin.

### Antibody blocking reduced L1-dependent cell adhesion and directional migration

**Figure 6:**
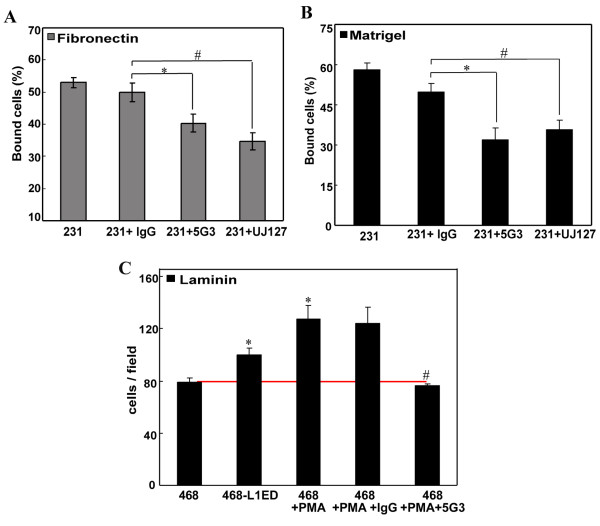
**Inhibition of cell adhesion and directional transmigration by L1CAM antibodies**. MDA-MB-231 cells pre-treated with control isotype IgG or either of the two anti-L1CAM monoclonal antibodies targeting its ectodomain (5G3 or UJ127) were added into 96-well plates coated with fibronectin (A) or Matrigel™ (B) for adhesion assay as described before. The antibody was also present during the incubation time. (C) Conditioned medium with 0.5% BGS from each cell culture system shown on X-axis was collected and added into the lower chamber as chemoattractant in transmigration assay. Where indicated, L1CAM antibody 5G3 or isotype IgG were added at 2 μg/mL in the lower chamber. MDA-MB-468 cells were seeded in serum free medium in the top and allowed to migrate for 12 hours. Migrant cells to the underside were stained and counted for 5 random fields to obtain the average. *, p < 0.05 vs. conditioned medium from MDA-MB-468; #, p < 0.05 vs. treatment with control antibody.

Monoclonal antibody (mAb) blocking has been reported as an efficient treatment for targeting specific proteins [24,37]. Here, we used two mAbs against different domains of the L1ED to examine the effect of blocking L1 in cell adhesion and motility assays. Compared to isotype IgG treatment, MDA-MB-231 cells blocked by L1 antibodies had significantly decreased binding ability to fibronectin (by 17%, Figure 6A) and to diluted Matrigel™ (by 25%, Figure 6B). Thus, monoclonal antibodies 5G3 and UJ127, which target different extracellular domains of L1, displayed almost no difference in their ability to block cell adhesion of MDA-MB-231 cells.

Purified L1-Fc has been reported as an adhesion substrate for ovarian cancer cells and attractant for endothelial cell migration [38]. We used conditioned culture medium from L1ED over-expressing cells to evaluate whether sL1 can direct breast cancer transmigration in our model. Shown in Figure 6C, conditioned medium collected from both L1ED over-expressing (468-L1) and PMA activated (468 + PMA) cells attracted significantly more MDA-MB-468 cells to transmigrate onto laminin, as compared to plain (468) medium. This chemoattractant effect could be totally neutralized by adding mAb 5G3 against the L1ED (Figure 6C), while no decrease was observed with IgG control. Thus, this demonstrates that the L1ED can induce directional movement of breast cancer cells in our transmigration assay.

## Discussion

From a neural recognition molecule in brain development to a potential marker in cancer progression, this *de novo *function of L1CAM has been uncovered in several cancer types [4,39]. We analyzed previously generated datasets of clinical samples and found that L1 expression levels were correlated with breast cancer stage progression and also were higher in more metastatic breast cancer cell lines that we used for our experiments here. We present new insights into how soluble L1 facilitates breast cancer cell motility by demonstrating that ectopic L1ED expression increased cell adhesion and migration in less migratory cell lines. In addition, we displayed a new function of shed L1 as an extracellular attractant in cancer cell migration.

Molecular profiling has been a great aid in sorting out potential markers for early cancer diagnosis. From previously established databases of breast cancer clinical samples or *in vitro *cultured cell lines, the L1CAM gene or the region in the X chromosome where it is located (Xq28) [40,41] has been found to be abnormally amplified in some cases [33,42,43]. Its over-expression also is correlated with progressing stages of cancer in patients (Figure 1A, [33]). Results from analysis of our cultured cell lines revealed a similar trend (Figure 1B and 1C), with the less migratory MDA-MB-468 cells expressing the lowest level of L1CAM protein. Our laboratory also found L1 is expressed in invasive and metastatic SUM149 breast cancer cell line (data not shown) and others have shown L1 expression in MDA-MB-231 and -435 cells as well [9]. Though the mechanism of how L1 is upregulated in metastatic tumors is still undefined, it is coincidently upregulated with ADAM10 as target genes of β-catenin/TCF signaling, with co-expression at the invasive front of colon carcinomas [44]. Effects of various growth factors in cancer tissues [45] also may contribute to the abnormal presence of L1CAM. Interestingly, analysis of 145 primary breast tumors and 51 breast cancer cell lines (including MDA-MB-231) in another study [46] did not show that the expression of L1CAM was correlated with genome copy number, suggesting that expression was not due to genomic aberrations (e.g. amplification). However, they did not give L1 expression results for individual cell lines. We believe L1 promotes breast cancer progression by its upregulated expression and coincident proteolysis by ADAM10 to release the soluble L1ED, which then exerts its autocrine/paracrine stimulatory effect.

L1 functions in the developing nervous system mostly as a cell surface adhesion/recognition protein by homophilic binding and heterophilic interaction with ECM and other migration guiding cues [15]. It can become endocytosed and can activate the intracellular MAP kinase pathway [47-49] to elicit its effects. Regulated proteolysis has been detected in developing mouse brain [9]. However, the presence of unregulated levels of soluble L1ED during nervous system development causes severe forms of L1 syndrome [12,13,18-20], and constitutive L1 shedding can favor cell migration from tumors [3,28]. Gutwein and colleagues [30] have reported that in the AR breast cancer cell line, L1 cleavage by ADAM10 can be detected to occur both in minute vesicles termed exosomes and at the cell surface to be released into the culture medium. These two forms of cleaved L1 both can be considered to be forms of soluble L1 (sL1). Our analysis corroborated their study on L1CAM localization in intracellular vesicles and cleavage at the membrane-proximal end in different breast cancer cell lines. Although Mechtersheimer et al. [9] found only a slightly detectable band at around 30 kDa to suggest L1 proteolysis in MDA-MB-231 and MDA-MB-435 cells in their study, this difference could be a result of different antibodies being used or different culture conditions. Alternatively, the cytoplasmic domain already may have been degraded (presumably by γ-secretase), which sometimes occurred in our breast cancer cell lines as well (data not shown). Consistent with this idea is that they were able to clearly demonstrate soluble L1ED in the cell culture supernatant from those cell lines [9].

sL1 thus generated has been reported in various cancers to be an active factor in angiogenesis [38], anti-apoptosis [50] and cell migration [36]. The mechanisms proposed for sL1 working in tumors can be categorized based on to which part a receptor would primarily bind. With an RGD motif on its sixth Ig domain, the sL1 retains similar roles as cell-surface L1 and still can recruit and bind to integrins such as αvβ3, αvβ5, and αvβ3 [9,29] to activate FAK and Src at focal adhesion complexes. The signal transduction initiated thereafter can stimulate cytoskeleton protein reassembly to generate directional membrane protrusions. On the other hand, sL1 also can be integrated into the immediate extracellular matrix [51] by binding to laminin or proteoglycan as a chemoattractant for any following cells to attach and adhere. Either way, sL1 provides flexible connections with the ECM for cells to mobilize [52]. Since cell migration speed potentially depends on the turnover rates of adhesion and de-adhesion cycles [53], fast and small focal complexes recruited by L1 binding and signaling actually facilitate the event of cell movement [54]. Our results obtained by overexpressing sL1 in MDA-MB-468 cells supported such a mechanism (Figures 3 and 4). Specifically, ectopic L1ED expression alone is sufficient to cause more cells to adhere to matrix and then to migrate. However, no significant change was observed in the Matrigel™ cell invasion assay. This suggests that soluble L1 serves mostly as a released factor to allow adhesion and exploration of the cell's immediate environment. In order to invade through the ECM, gene expression changes causing proteolysis and degradation of the matrix are required [54,55], which evidently are not achieved by adding sL1 alone in a short time period.

Given that L1CAM is rarely present other than in the nervous system, immune effector cells, and kidney under normal circumstances [23,41], its constitutive expression and shedding in tumors makes it an ideal marker for cancer detection and treatment. For example, short hairpin RNA targeting L1CAM, which has been found to impair axon outgrowth [56] in normal neurons, can disrupt cell proliferation and neurosphere formation of brain tumor cells [57]. Our lab has found that attenuating L1CAM by this method in glioma cells decreased focal complex turnover, reduced cell motility *in vitro*, and halted brain invasiveness *in vivo *[32]. Here, we showed that shRNA targeting L1 in breast cancer cells can weaken cell adhesion and transmigration ability to a significant extent. Also, antibodies blocking L1, which have been used by others [24] and us [32,58] to inhibit cancer cell growth and motility, were shown to reduce breast cancer cell adhesion and migration in our assay. These results point to L1CAM as being an over-expressed cell surface molecule in tumors and that it contributes importantly to cell migratory behavior.

PMA is reported to elevate L1 shedding by activating the PKC pathway [59]. In our experiments, PMA treatment increased sL1 in the culture medium, whereas the overall L1 protein expression level remained unchanged. This could be because activation of the MAPK network upon addition of PMA initiated gene expression [60,61], but did not increase L1CAM expression directly. Whereas, the expression level of ADAM10 or another protease involved in ADAM maturation [62] may be promoted, so that more surface L1, together with other protein substrates, would be cleaved. For the same reason, more MDA-MB-468 cells migrated toward conditioned medium from PMA activated cells. In that analysis, it is hypothesized that soluble L1 in conditioned medium might have bound to the coating matrix like integrated tracks in the ECM, attracting the cells to migrate and/or L1 could have exerted its effects in its soluble form. Additionally, homophilically-bound L1 fragments might have caused integrin recruitment on cell protrusions to form focal complexes and speed up the overall motility [53]. The reason why supernatant from PMA-activated cells is more attractive than supernatant from 468-L1ED cells could be because more active cell responses were initiated via the PKC pathway stimulated by PMA. Nonetheless, the dramatic increase in transmigration could be totally reversed by addition of anti-L1CAM antibody (Figure 6C), demonstrating that the attractant was the L1ED.

## Conclusions

Overall, our present study demonstrates a positive correlation of L1 expression level with breast cancer cell migratory ability. The role of L1CAM and its soluble form was shown to facilitate cell adhesion to ECM and transmigration ability by experimentally increasing the soluble L1ED as well as attenuating L1 protein levels. sL1 also was shown to be an attractant for directional breast cancer cell migration. These effects of over expression of the L1ED in low migratory breast cancer cells (MDA-MB-468) parallel the role of L1 in early brain development, however in an unregulated manner. Consequently, abnormal L1CAM expression may be a good marker for detection of breast cancer progression and metastatic potential.

## Methods

### Cell culture conditions

Human breast cancer cell lines MDA-MB-231, MDA-MB-435 and MDA-MB-468 were used. MDA-MB-231 (ATCC No. HTB-26) was a gift from Dr. Ulhas Naik (Univ. of Delaware), MDA-MB-435 cell line was gifted by Dr. Danny Welch (Univ. of Alabama, Birmingham) and MDA-MB-468 cells were from Dr. Leslie Krueger (A.I. DuPont Hospital for Children). These three cell lines were maintained in DMEM (Mediatech Inc., Herndon, VA), supplemented with 10% bovine growth serum (BGS; Hyclone, Waltham, MA), 100 μg/mL penicillin/streptomycin (Mediatech Inc.) and 2 mM L-glutamine (Mediatech Inc.). Quail fibrosarcoma cell line QT6 cells were cultured in Medium 199 (Mediatech, Inc.) with 5% FBS, 20% tryptose phosphate broth, 2 mM L-glutamine and penicillin-streptomycin. Human glioma cell line U-87 MG was obtained and kept as described in [36]. All cell lines were cultured at 37°C in a humidified atmosphere containing 5% CO2.

### Antibodies and reagents

Three different antibodies against L1CAM were used. Polyclonal antibody NCAM-L1 (C-20) (sc-1508, Santa Cruz Biotechnology, Santa Cruz, CA) recognizes the C-terminal region of L1CAM protein, monoclonal antibody 5G3 (sc-33686, Santa Cruz Biotechnology) recognizes the first Ig domain and UJ127 recognizes the 5^th ^fibronectin repeat (GTX23200; Gene Tex, Irvine, CA). Monoclonal antibody against β-tubulin (Developmental Studies Hybridoma Bank, Iowa City, Iowa) and mouse IgG whole molecule (Jackson ImmunoResearch, West Grove, PA) were used for controls where indicated. Phorbol 12-myristate 13-acetate (PMA) was purchased from Sigma (Sigma-Aldrich Corp., St. Louis, MO).

### RT-PCR analysis

RNA extraction and generation of first strand cDNA was carried out using PureLink total RNA purification system (Invitrogen, Carlsbad, CA) and the SuperScript III First-Strand Synthesis System (Invitrogen). PCR was performed using the following primers with the length for each product indicated in parenthesis:

L1CAM [GeneBank: 3897] sense, 5'-TACCGCTTCCAGCTTCAG -3';

antisense, 5'- TGATGAAGCAGAGGATGAGC -3' (460bp)

-actin [GeneBank: 60]: sense, 5'- GCTCGTCGTCGACAACGGCTC -3';

antisense, 5'- CAAACATGATCTGGGTCATCTTCTC-3' (353bp).

PCR was carried out as described [32] using Master Mix (Promega, Madison, WI) in a thermal cycler (Techne, Burlington, NJ), and products were then electrophoresed on a 1.5% agarose gel and visualized by ethidium bromide staining.

### Vector construction and lentivirus infection

L1 ectodomain (L1ED) fragment was generated from the pCDNA3-L1 vector kindly provided by Dr. Vance Lemmon (The Miami Project to Cure Paralysis, University of Miami, FL). The following primers with SpeI or XhoI (NEB, Ipswich, MA) cleavage sites were used to amplify the sequence: sense, 5'-GAAACTAGTCGCCGGGAAAG-3'; antisense, 5'- GCCTCGAGGAGGGAGCC- 3'. The resulting 3350bp L1ED was then inserted into a lentivirus vector (Lvv 1879; provided by Dr. John C. Kappes, Univ. of Alabama, Birmingham) containing a CMV promoter. Empty Lvv 1879 vector served as a negative control. Constructs obtained were both confirmed by DNA sequencing and used to make virus to infect MDA-MB-468 cells. MDA-MB-231 cells were infected with L1-shRNA vector or the mock control. An shRNA Lentiviral vector targeting human L1CAM (TRCN0000063917; cat No. RHS3979-97052304) and the non-targeting control vector pLKO.1 were purchased from Open Biosystem (Huntsville, AL). All four vector constructs were then transfected into HEK 293T/17 respectively, with the helper plasmid pMD.G and packaging plasmid pCMVΔR8.2 by ratio of 4:3:1 (20 μg: 15 μg: 5 μg for a 10 cm plate) using standard calcium phosphate method [63]. Supernatants containing viruses were collected 48 and 72 hours after transfection and target cells were infected using 10 μg/mL polybrene. Stable infected cell lines with encoded vectors were selected by 2 μg/mL puromycin resistance and confirmed by immunoblotting or FACS analysis.

### Western blot analysis

Protein extraction and western blot analysis was performed as described [36]. In brief, plain MDA-MB-231, MDA-MB-435 and MDA-MB-468 cells or those stably infected cell lines were solubilized using RIPA buffer. Cell lysates were then quantified using the BCA Assay (Pierce Biotechnology, Pittsburgh, PA). Unless otherwise indicated, a total of 30 μg target proteins for each sample were probed with antibodies as indicated in each test.

### Immunohistochemistry

Immunofluorescent staining was performed as before [36] with minor differences. In brief, cells cultured on coverslips pre-coated with 200 μg/mL poly-L-ornithine (Sigma-Aldrich, St. Louis, MO) were directly stained with primary antibodies diluted in PBS with 5% heat-inactivated serum on ice for live staining. Alternatively, cells were fixed with 1% formaldehyde in PBS followed by primary antibody diluted in PBS with 5% normal goat serum and 0.03% Triton-X 100. In either case, the coverslips were then rinsed with PBS and incubated with Alexa Flour 594 secondary antibody (Molecular Probes, Invitrogen) diluted in PBS with 5% normal goat serum for 45 min-1 hr at room temperature. After washing, nuclei were stained in 10 μg/mL bisbenzimide (Sigma-Aldrich) and mounted. Thus prepared slides were visualized and digital images were taken by using a Zeiss LSM 510 Confocal imaging system (Carl Zeiss, Thornwood, NY, USA) with appropriate argon beam lasers.

### FACS analysis

Cells were trypsinized, fixed (as described above) and stained with a saturating amount of mAb UJ127 or just secondary antibody Alexa Fluor-488 (Molecular Probes, Invitrogen) as control. Stained cells were examined and analyzed using a FACSCalibur flow cytometer (Becton Dickinson) using Cell Quest software.

### L1 shedding analysis

L1 shedding analysis was performed as described [9] with minor modifications. In brief, 10^6 ^cells were cultured in 35 mm tissue culture plates in complete medium for 24 hrs. The next day, culture plates were rinsed with PBS to remove growth factors and then 1 mL serum-free DMEM was replaced for an hour while cells remained normally attached. Where indicated, PMA (100 ng/mL) was co-incubated with the cells. After 1 hr incubation at 37°C, culture medium was separated from cell debris by centrifugation. The culture media were TCA precipitated and dissolved in LDS loading buffer (Invitrogen), while the pellets were lysed respectively as in western blot analysis. Proteins thus obtained were all subjected to western blotting for the presence of L1 with antibodies as indicated.

### Cell adhesion assay

Cell adhesion assays were performed in 96-well flat bottom plates coated with different ECM proteins. Briefly, 50 μL/well solution of either fibronectin (10 μg/mL; Sigma-Aldrich) or laminin (10 μg/mL; Invitrogen) in PBS, or Matrigel™ (20 μg/mL; BD) in serum free DMEM were pre-added to 96-well plates and incubated at 4°C overnight. The plates were then rinsed and blocked with 0.2% BSA for 2 h at room temperature followed by three times PBS washing. Cells were then added to each well in triplicate and incubated for 45 min at 37°C. After washing, cells remaining attached to the plates were fixed and stained with a solution containing 0.5% crystal violet, 2% ethanol and 40% methanol in PBS. 100 μL SDS (1% wt/vol) was added to each well after washing, and the absorbance of the color substrate was measured with an FLUOstar OPTIMA microplate reader (BMG Labtechnologies, Offenburg Germany) at 595 nm. The percentage of bound cells was calculated by dividing the optical density of the adherent cells by that of the initial input cells, with BSA-coated wells subtracted as background reading. Where indicated, mAbs or mouse IgG negative controls were pre-incubated with the cells for 30 min at 4°C and then kept present during the adhesion period.

### Cell transmigration and invasion assay

Cell transmigration assays were performed as described [9] using 8.0 μm pore size Transwell inserts (Costar, Cambridge, MA) with some modifications. In brief, the bottom of the insert membrane was coated with either fibronectin or laminin at 10 μg/mL in PBS at 37°C for 90 min or 4°C overnight. Cells were serum-starved overnight (0.5% FBS), harvested with trypsin, washed and then resuspended in basal DMEM medium without serum. 10^5 ^cells in 0.1 mL were then added to the upper chamber of the inserts, and 0.6 mL DMEM with 10% serum was added to the lower chamber. Where indicated, conditioned medium obtained as described [64] from cell culture supernatants was used instead in the lower chamber. After 12 h at 37°C, cells remaining on the upper side of the membrane were removed using cotton swabs, while the cells that migrated to the underside were fixed in methanol at room temperature for 30 min and then stained with crystal violet solution. The number of migrated cells was counted under a microscope in five fields at 100× magnification. Each assay was done in triplicate and presented as mean ± SEM. Cell invasion assays were done similarly to transmigration assays, only instead, transwell inserts were pre-coated with Matrigel™ at 1 μg/μL on the topside. Cells were allowed to invade for 24-48 hrs under normal culture conditions.

### Statistical Methods

Data presented are mean ± SEM of at least three repeats. Student's t-test was used to analyze difference between two groups. ANOVA was used when more than two groups were involved, and then Student's t-test was further applied to analyze difference between groups. * or #, P < 0.05 was considered as significant; **, P < 0.01.

## List of Abbreviations

ADAM10: A Disintegrin and Metalloprotease 10; CAM: Cell Adhesion Molecules; ECM: Extracellular Matrix; FACS: Flow Cytometry; FN: fibronectin; FN Repeats: Fibronectin-like Repeats; Ig Domains: Immunoglobulin-like Domains; L1ED: L1 Ecto-Domain; LN: Laminin; Lvv: Lentiviral Vector; PMA: Phorbol 12-myristate 13-acetate; RGD: Arg-Gly-Asp; RT-PCR: reverse transcriptase-polymerase chain reaction; sL1 Soluble L1; TCA: Trichloroacetic acid; 231: MDA-MB-231; 435: MDA-Mb-435; 468: MDA-MB-468.

## Competing interests

The authors declare that they have no competing interests.

## Authors' contributions

YL and DSG designed experiments, conducted experiments and analyzed data. YL and DSG drafted and edited the manuscript. Both authors read and approved the final manuscript.
